# *Geobacter sulfurreducens* metabolism at different donor/acceptor ratios

**DOI:** 10.1002/mbo3.1322

**Published:** 2022-09-22

**Authors:** Hanna Marianne Frühauf‐Wyllie, Dirk Holtmann

**Affiliations:** ^1^ Department of Chemical Technology DECHEMA Forschungsinstitut Frankfurt am Main Germany; ^2^ Technische Hochschule Mittelhessen, Institut für Bioverfahrenstechnik und Pharmazeutische Technologie Gießen Germany

**Keywords:** central metabolism, donor/acceptor ratio, *Geobacter sulfurreducens*, organic acid production

## Abstract

*Geobacter* species have great application potential in remediation processes and electrobiotechnology. In all applications, understanding the metabolism will enable target‐oriented optimization of the processes. The typical electron donor and carbon source of the *Geobacter* species is acetate, while fumarate is the usual electron acceptor. Here, we could show that depending on the donor/acceptor ratio in batch cultivation of *Geobacter sulfurreducens* different product patterns occur. With a donor/acceptor ratio of 1:2.5 malate accumulated as an intermediate product but was metabolized to succinate subsequently. At the end of the cultivation, the ratio of fumarate consumed and succinate produced was approximately 1:1. When fumarate was added in excess, malate accumulated in the fermentation broth without further metabolization. After the addition of acetate to stationary cells, malate concentration decreased immediately and additional succinate was synthesized. Finally, it was shown that also resting cells of *G. sulfurreducens* could efficiently convert fumarate to malate without an additional electron donor. Overall, it was demonstrated that by altering the donor/acceptor ratio, targeted optimization of the metabolite conversion by *G. sulfurreducens* can be realized.

## Graphical abstract

The usual electron donor and carbon source of the *Geobacter* species is acetate, while fumarate is the usual electron acceptor. By choosing different acceptor/donor ratios, malate and succinate production by *Geobacter sulfurreducens* can be altered. The results provide optimization possibilities for chemical synthesis as well as for the application of *G. sulfurreducens* in electrobiotechnology and remediation processes.

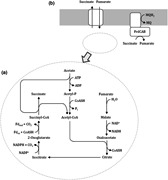

## 1 INTRODUCTION

*Geobacter* strains are deltaproteobacteria and can be found in different habitats, for example, aquatic systems, deep aquifer sediments, subsurface sediments, as well as several contaminated sites. *Geobacter* cells are Gram‐negative, nonspore‐forming, and obligate heterotrophs (Straub, 2011; Zhang et al., 2020). In general, *Geobacter* species grow only under strictly anoxic conditions and are unable to grow by fermentation (Straub, 2011). *Geobacter* species predominate under iron‐reducing conditions (Straub, 2011). Acetate is a common electron and carbon donor among *Geobacter* species (Straub, 2011) and the majority of species could also metabolize alternative electron donors (e.g., ethanol, pyruvate, lactate, hydrogen, and formate). *Geobacter* species are using different electron acceptors, for example, Fe(III), nitrate, elemental sulfur (S^0^), fumarate, malate, Mn(IV), U(VI), Co(III), and humic substances. *Geobacter* species appear to be the primary agents for coupling the oxidation of organic compounds to the reduction of insoluble Fe(III) and Mn(IV) oxides in many soils and sediments (Lovley et al., 2011). Different G*eobacter* species can oxidize aromatic hydrocarbons under anaerobic conditions. In addition, *Geobacter* species are important for aromatic hydrocarbon removal in contaminated aquifers (Lovley et al., 2011). Furthermore, different *Geobacter* species can transfer electrons from the metabolism oxidation of organic compounds to an electrode. Direct interspecies electron exchange between *Geobacter* species and syntrophic partners appears to be an important process in anaerobic wastewater treatment (Lovley et al., 2011).

**Figure 1:**
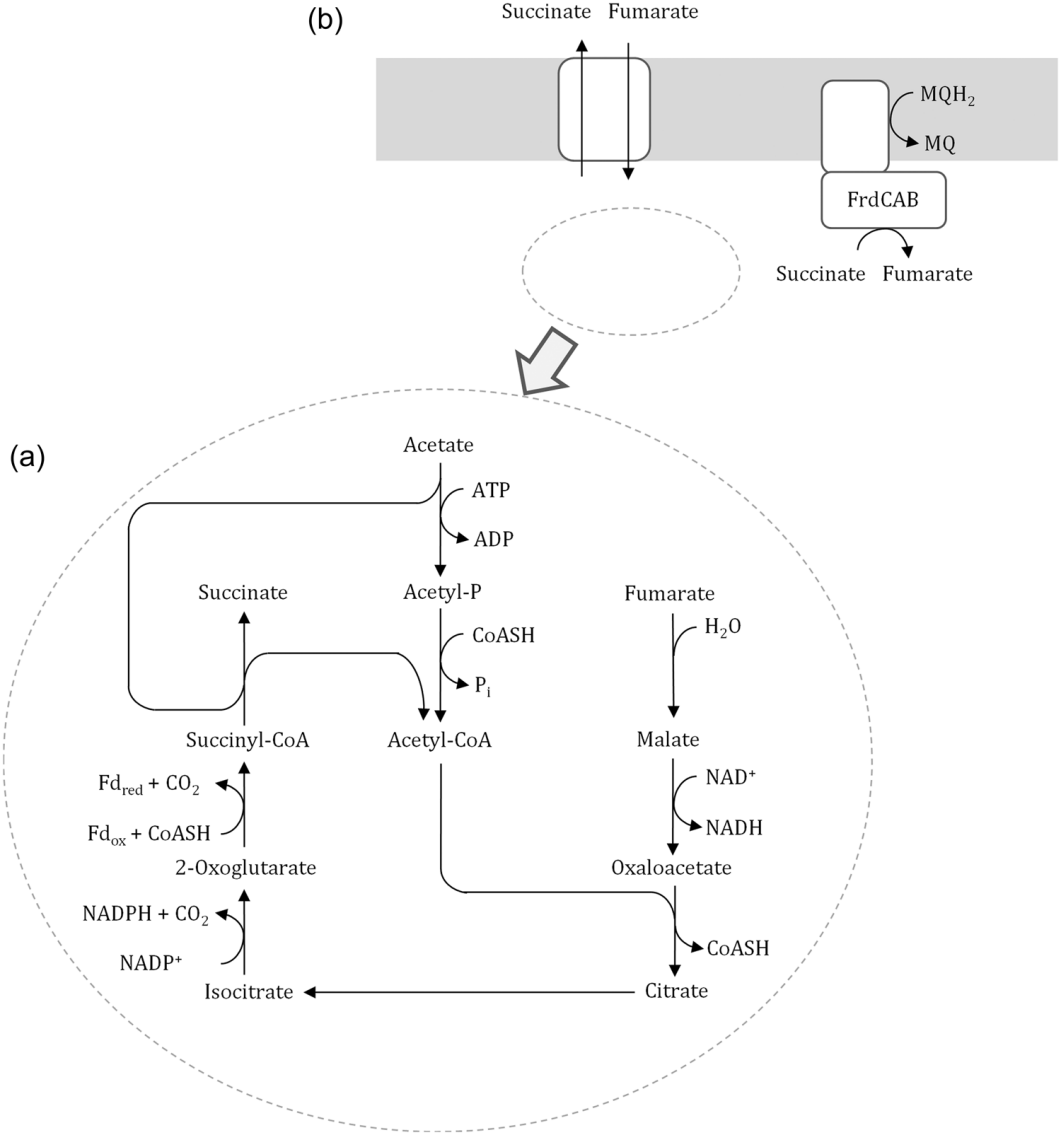
(a) Acetate conversion in the TCA cycle with fumarate as electron acceptor. TCA is not closed as succinate is secreted to the medium and external fumarate is continuously supplied to fuel the reaction (adapted from Galushko & Schink, 2000). (b) Fumarate is simultaneously reduced as input to the TCA and by FrdCAB, which is coupled to ATP synthesis via the menaquinone pool (adapted from Butler et al., 2006). TCA, tricarboxylic acid cycle, NAD/NADH, reduced/oxidized nicotinamide adenine dinucleotide, NAPD/NADPH, reduced/oxidized nicotinamide adenine dinucleotide phosphate

*Geobacter sulfurreducens* has served as a model organism as it is amenable to genetic manipulation and was the first *Geobacter* species to have its genome fully sequenced (Methé et al., 2003; Tabares et al., 2020). *G. sulfurreducens* can grow on acetate, H_2_, lactate, formate, and CO as electron donors (Geelhoed et al., 2016; Lovley et al., 2011; Speers Allison & Reguera, 2012), typical electron acceptors are Fe(III)‐citrate, Fe(III)‐phosphate, Co(III), U(VI), S^0^, fumarate, malate (Lovley et al., 2011) and to some extent also O_2_ (microaerobic growth reported in (Engel et al., 2020; Lin et al., 2004). This article aims to investigate the donor/acceptor‐pair acetate‐fumarate in more detail. Here, acetate is oxidized to CO_2_ via the tricarboxylic acid cycle (TCA), while fumarate is reduced to succinate (Equation (1a), (1b) and Figure 1, Galushko & Schink, 2000).

(1a) $${\text{CH}}_{3}{\text{COO}}^{-}+4C_{4}H_{2}{O_{4}}^{2-}+2H_{2}O+H^{+}\to 2{\text{CO}}_{2}+4C_{4}H_{4}O_{4}^{2-},$$

(1b) $${\text{Acetate}\;}^{-}\;+\;4{\text{Fumarate}}^{-}\;+\;2H_{2}O+H^{+}\to 2{\text{CO}}_{2}+4\text{Succinate}$$

The complete oxidation of 1 mol acetate releases 8 mol electrons, thus up to 4 mol of fumarate can be reduced to 4 mol succinate for every mol acetate. In growing cells, approximately 50% of the consumed acetate is dissimilated and the remaining acetate is used for cell synthesis (Galushko & Schink, 2000). Hence, the actual donor/acceptor ratio observed is approximately 1:2. Fumarate is entirely reduced to succinate, which is secreted to the medium, so the TCA as found in *G. sulfurreducens* metabolism is not closed. Instead, externally added fumarate is converted to oxaloacetate by fumarase and malate dehydrogenase and continuously introduced to the TCA cycle (Galushko & Schink, 2000). Additionally, fumarate is reduced at the membrane‐bound *G. sulfurreducens* fumarate reductase FrdCAB which is coupled to the menaquinone pool and thereby to ATP synthesis (Butler et al., 2006). This enzyme simultaneously acts as succinate dehydrogenase to close the TCA cycle when not fumarate but Fe(III) is the electron acceptor (Butler et al., 2006; Esteve‐Núñez et al., 2005). ATP is solely synthesized by electron transport phosphorylation, fueled by NADH and NADPH delivered to the menaquinone pool (Galushko & Schink, 2000). Fumarate is frequently used as a soluble terminal electron acceptor in *G. sulfurreducens* growth medium but is usually omitted in microbial fuel cell experiments to channel electrons exclusively to the electrode.

Malate can be used in different technical applications, for example, in the food and beverage industry, chemical synthesis, textile finishing, and pharmaceutical industries (Jiang et al., 2020; Kövilein et al., 2020). Besides the chemical synthesis by hydration of maleic anhydride generated from the oxidation of benzene or butane, malate or maleic acid can be produced enzymatically by using the fumarase activity or microbial synthesis from renewable substrates (Jiang et al., 2020). Data regarding the market volume of malic acid range between 60,000 and 200,000 tons per year (Kövilein et al., 2020). Succinate or 1,4‐butanedioic acid is a four‐carbon dicarboxylic acid. Besides different applications in the food industry, succinate is a precursor for the production of many high‐value chemicals, for example, 1,4‐butanediol, tetrahydrofuran, and polybutylene succinate. Due to its versatile applications, succinate is rising to a bulk chemical in recent years. Its annual global production is estimated at between 30,000 and 50,000 tons (adapted from Cao et al., 2013).

In the following experiments, acetate/fumarate conversion of *G. sulfurreducens* was studied by varying donor/acceptor ratios. As mentioned above, the theoretical optimum ratio between acetate as donor and fumarate as acceptor is 1:2. In contrast, the ratio proposed in the often applied DSMZ medium recipe is 1:5. Therefore, in the present study, growth and metabolism were monitored at different ratios and by using growing and resting (stationary) cells. In all experiments, the resulting kinetics as well stoichiometry were evaluated.

## 2 MATERIALS AND METHODS

All chemicals were of at least analytical grade and purchased from Roth, Sigma‐Aldrich, and Fluka.

### 2.1 Strains and culture/growth conditions

All methods are described in detail in (Frühauf et al., 2022; Frühauf et al., 2022) and are only briefly described here. *G. sulfurreducens* strain PCA (DSM 12127) was obtained from DSMZ (German Collection of Microorganisms and Cell Cultures GmbH). All cultivations were done anaerobically in serum flasks sealed with a butyl septum (Glasgerätebau Ochs). Flasks were incubated shaking at 30°C and 180 rpm (Shaking throw 25 mm, Ecotron Infors HT shaker). The standard growth medium was DSM 826 and contained (per liter): 0.1 g KCl, 1.5 g NH_4_Cl, 0.5 g Na_2_HPO_4_, 0.82 g Na‐Acetate as electron donor, 4.8 g Na‐fumarate as an electron acceptor, 2.5 g NaHCO_3_, 10 ml of vitamin mix, and 10 ml of trace mineral mix. Vitamin mix contained (per liter): 2 mg biotin; 2 mg folic acid; 10 mg pyridoxine‐HCl; 5 mg thiamine‐HCl × 2 H_2_O; 5 mg riboflavin; 5 mg nicotinic acid; 5 d‐Ca‐pantothenate; 0.1 mg vitamin B_12_; 5 mg *p*‐aminobenzoic acid, and 5 mg lipoic acid. Trace element solution contained: 1.5 g nitrilotriacetic acid; 3 g MgSO_4_ × 7 H_2_O; 0.5 g MnSO_4_ × H_2_O; 1 g NaCl; 0.1 g FeSO_4_ × 7 H_2_O; 0.18 g CoSO_4_ × 7 H_2_O; 0.1 g CaCl_2_ × 2 H_2_O; 0.18 g ZnSO_4_ × 7 H_2_O; 0.01 g CuSO_4_ × 5 H_2_O; 0.02 g KAl(SO_4_)_2_ × 12 H_2_O; 0.01 g H_3_BO_3_; 0.01 g NaMoO_4_ × 2 H_2_O; 0.03 g NiCl_2_ × 6 H_2_O; 0.30 mg Na_2_SeO_3_ × 5 H_2_O; 0.40 mg Na_2_WO_4_ × 2 H_2_O). The medium containing all components except for fumarate, NaHCO_3,_ and vitamin solution was degassed with N_2_/CO_2_ (80/20) (Aligal 12™; Air Liquide) gas mixture for 90 min, afterward, NaHCO_3_ was added and the medium transferred to an anaerobic chamber (Rigid Chamber, Coy Laboratory Products Inc.). Each 48 ml medium was aliquoted under N_2_/H_2_ (95/5) atmosphere (forming gas) to 250 ml serum flasks, sealed with a butyl septum, and the septum secured with aluminum caps (Glasgerätebau Ochs). Then the forming gas atmosphere was exchanged by evacuating the flasks three times and refilling them with N_2_/CO_2_ gas mixture. Subsequently, serum flasks were autoclaved. Before microbial cultivation 1.5 ml Na‐fumarate (160 g/L) (if not indicated differently) and 0.5 ml vitamin solution were added to each flask and degassed for another 15 min to remove any oxygen that might have diffused through the septum during storage of the flask. The final pressure in the septum flask was 1.8 bar. Before inoculation, medium and inoculum were prewarmed for 30 min and 1.5 ml of a stationary culture (maintenance culture) was used to inoculate a fresh culture. The maintenance culture was stored for a maximum of 2 weeks in the dark at 4°C and refreshed every 2 weeks from a cryo culture.

### 2.2 Analytics

Growth experiments were carried out in triplicates and monitored by measuring OD_600_ and metabolite concentration (acetate, fumarate, malate, and succinate) in the supernatant using HPLC analysis. Growth and metabolite kinetics were calculated using the R package “growthcurver,” which fits the optical density or concentration data to a logistic equation and allows the estimation of growth rate, respectively, production/consumption rate, as well as doubling time and other growth parameters (Sprouffske & Wagner, 2016). For sampling, shaking flasks were always transferred to the anaerobic chamber to avoid oxygen entering the flask when drawing a sample. For each sample, 0.8 ml samples were drawn with a syringe and transferred to a cuvette to measure OD_600_. Afterward, the sample was filtered with a 0.2 μm polyvinylidene fluoride filter and transferred to an HPLC vial. Samples were stored at −20°C until further use.

Acetate, fumarate, malate, and succinate concentration was determined in an HPLC (Shimadzu Deutschland GmbH), using a Rezex™ ROA‐Organic Acid H+ (8%) column (300 × 7.8 mm) with a SecurityGuard Standard Carbo H + cartridge (4 × 3 mm, both Phenomenex Ltd.), with a refractive index detector (RID‐10A). Column method was 5 mM H_2_SO_4_, 0.6 ml/min, 30°C, 24 min. Concentrations of calibration standards for all components (Na‐Acetate, Na_2_‐fumarate, DL‐malic acid, and succinic acid) ranged from 0.5 to 100 mM and a calibration curve was determined separately for each new HPLC measurement. Retention times: acetate: 16.7 min; fumarate: 17.6 min; malate: 11.4 min; succinate: 13.9 min.

## 3 RESULTS AND DISCUSSION

**Figure 2:**
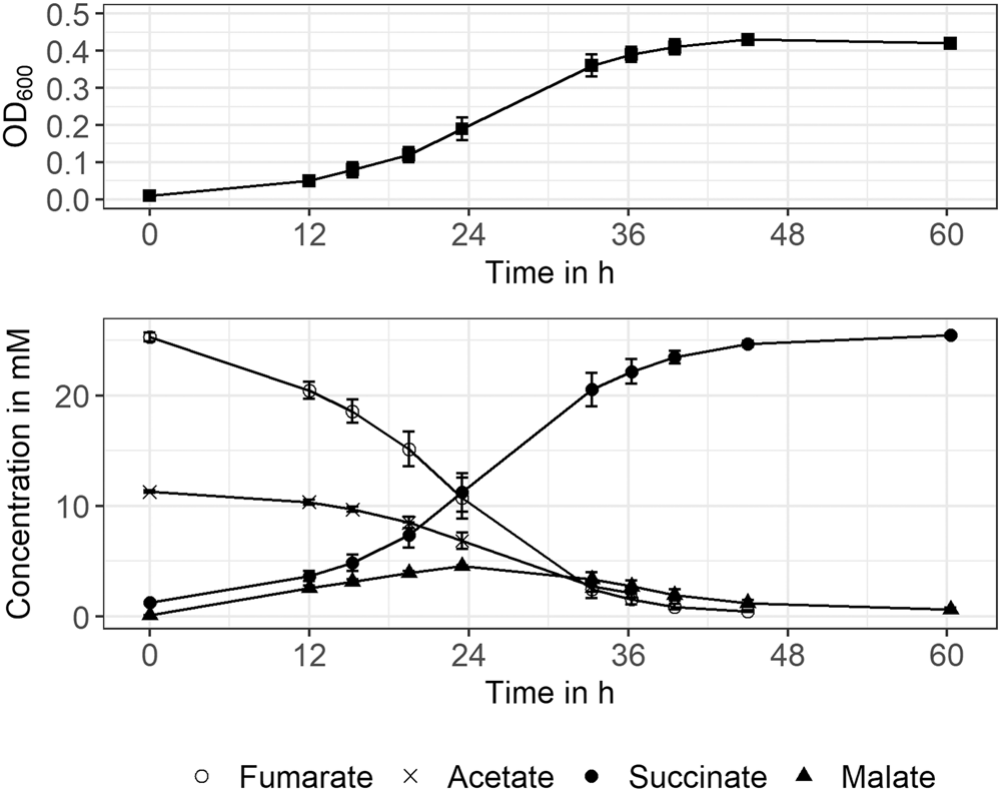
Mean values for OD_600_ and metabolite concentration are shown over time when cultivating *Geobacter sulfurreducens* with 10 mM acetate and 25 mM fumarate. Error bars show SD for *n* = 3.

Growth curves of a biological triplicate with 10 mM acetate and 25 mM fumarate were monitored over 60 h. The growth curve and metabolite concentrations are shown in Figure 2. With an acceptor/donor ratio of 1:2.5, the growth rate was 0.19 ± 0.05 h^−1^, with a maximum calculated doubling time of 3.69 ± 0.06 h. This doubling time is significantly lower compared to the doubling time calculated in Galushko and Schink (2000) (7.7 h) but it has to be noted that in the work by Galushko and Schink, different medium and unshaken cultures were used, which might reduce nutrient availability and thereby growth rate. With the initiation of the exponential growth phase, fumarate is consumed at a linear rate of −0.87 ± 0.01 mM h^−1^. At the same time, succinate concentration increases at an exponential rate of 1.09 ± 0.003 mM h^−1^. The carbon and electron source acetate is consumed at −0.36 ± 0.02 mM h^−^^1^. Fumarate is consumed faster than acetate even though acetate is the only electron source for fumarate reduction. Together with succinate as the product of fumarate reduction, malate is produced as an intermediate. Its maximum concentration is reached after approximately 24 h of cultivation; until then, the linear production rate is 0.19 ± 0.003 mM h^−^^1^. Afterward, malate is consumed and metabolized to the final product succinate via the citric cycle. At the end of the cultivation, the ratio of fumarate consumed and succinate produced is 1:1.

**Figure 3:**
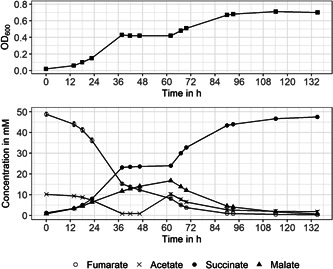
Mean values for OD_600_ and metabolite concentration are shown over time when cultivating *Geobacter sulfurreducens* with excess fumarate. Error bars show SD for *n* = 3. 10 mM acetate was added to the stationary culture after 60 h of cultivation.

In the following, the donor/acceptor ratio was altered to 1:5. When fumarate is added in excess, malate accumulation is faster with a rate of 0.28 ± 0.01 h^−^^1^ and to a maximum concentration of 17 mM, in comparison to 5 mM when only 25 mM fumarate is available initially. Also, malate was accumulated continuously and not transiently (Figure 3). After the culture reached the stationary growth phase, 10 mM acetate was added to monitor malate uptake by the cells when the electron source is refilled. The first growth phase (until 60 h) follows logistic growth as seen in Figure 3, the one after fresh acetate is fed seems to follow a limited growth model. The growth rate for the first growth term is 0.23 ± 0.01 h^−^^1^ with a maximum calculated doubling time of 3.05 ± 0.09 h^−^^1^, which is slightly faster than growth with a 25 mM electron acceptor. Acetate and succinate metabolization were at similar rates compared to growth with a 1:2.5 donor/acceptor ratio, also showing that excess fumarate concentrations do not inhibit growth. Fumarate is metabolized at a faster rate with −1.23 ± 0.03 h^−^^1^, which correlates with the faster malate accumulation. When acetate was available again, malate concentration decreased immediately and cell growth resumed, but only to 80% of the OD_600_ that was expected possible with 20 mM acetate in total (theoretical OD_600_ circa 0.84, actual OD_600_ 0.7).

**Figure 4:**
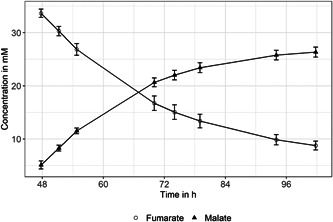
Conversion of 35 mM fumarate by stationary cells of *Geobacter sulfurreducens*. Error bars show SD for *n* = 3.

To test the activity of fumarase and malate transporter of resting cells 35 mM fumarate was added to a stationary culture (OD_600_ 0.43) and incubated without carbon or electron source. In Figure 4, the conversion of fumarate to malate can be seen, following a classical limited conversion model, shown in Equation (2) with *x* as time in hours and parameter *b* positive for fumarate concentration kinetics and negative for malate kinetics. With 0.047 ± 0.004 mM h^−^^1^, the consumption rate of fumarate approximately equals malate production (−0.050 ± 0.005 mM h^−^^1^). In 54 h, 25 mM fumarate is converted to 21 mM malate, which equals an 85% conversion before saturation is reached. The slight discrepancy to a full conversion might primarily be caused by remaining acetate, stored inside the cells, which serves as an electron source to metabolize malate further, and additionally by deviations in HPLC analysis. Nevertheless, the analyzed conversion rate is in the range of typical values (Kövilein et al., 2020). The continuous depletion of fumarate indicates that fumarase acts independently of the following malate conversion towards the TCA and that the malate concentration in the medium should be the primary indicator when assessing whether soluble electron acceptor is available for the organism.

(2) f(x)=a+b·e(−k·x).

When fumarate as a soluble electron acceptor is available in excess, it is constantly converted to malate, even by resting cells. Transient malate accumulation was also observed in Butler et al. (2006) and Galushko and Schink (2000) and explained by the thermodynamically unfavorable oxidation from malate to oxaloacetate. To still shift the equilibrium toward oxaloacetate, malate is accumulated by the faster reaction of fumarase, converting fumarate to malate (Galushko & Schink, 2000). This effect is more pronounced the higher the fumarate excess. In chemostat studies in vivo flux analysis showed that when fumarate was used as the electron acceptor, fumarate was not only reduced to succinate but also converted to malate by fumarase and further to oxaloacetate via malate dehydrogenase (Yang et al., 2010). The malate dehydrogenase activity seems to be one limiting step in the conversion of the bioconversion using the redox pair acetate/fumarate (Muhamadali et al., 2015). In summary, the results underline that by using different acceptor/donor ratios, malate and succinate production by *G. sulfurreducens* can be altered specifically. The results expand our knowledge of *G. sulfurreducens* metabolism and provide optimization possibilities for chemical synthesis as well as for the application of *G. sulfurreducens* in electro‐biotechnology and in remediation processes.

## AUTHOR CONTRIBUTIONS

**Hanna Marianne Frühauf‐Wyllie**: Conceptualization (equal); data curation (equal); investigation (lead); methodology (lead); writing – original draft (equal); writing – review and editing (equal). **Dirk Holtmann**: Conceptualization (equal); data curation (equal); funding acquisition (lead); project administration (lead); writing – original draft (equal); writing – review and editing (equal).

## CONFLICT OF INTERESTS

None declared.

## ETHICS STATEMENT

None required.

## Data Availability

All data generated and analyzed during this study are included in this published article.
